# The longitudinal association between the use of antihypertensive medications and 24-hour sleep in nursing homes: results from the randomized controlled COSMOS trial

**DOI:** 10.1186/s12877-021-02317-4

**Published:** 2021-07-18

**Authors:** Elisabeth Flo-Groeneboom, Tony Elvegaard, Christine Gulla, Bettina S Husebo

**Affiliations:** 1 Centre for Elderly and Nursing Home Medicine, Department of Global Public Health and Primary Care, University of Bergen, Bergen, Norway; 2 Department of Clinical Psychology, University of Bergen, Postboks 7807, 5020 Bergen, Norway; 3 National Centre for Emergency Primary Health Care, NORCE Norwegian Research Centre, Bergen, Norway; 4 Municipality of Bergen, Bergen, Norway

**Keywords:** Sleep, Actigraphy, Blood pressure, Antihypertensives, Antihypertensive medication use

## Abstract

**Background:**

Antihypertensive medication use and sleep problems are highly prevalent in nursing home patients. While it is hypothesized that blood pressure and antihypertensive medication use can affect sleep, this has not been investigated in depth in this population. Alongside a multicomponent intervention including a systematic medication review, we aimed to investigate the longitudinal association between antihypertensive medication use, blood pressure and day- and night-time sleep over 4 months.

**Methods:**

This study was based on secondary analyses from the multicomponent cluster randomized controlled COSMOS trial, in which the acronym denotes the intervention: COmmuncation, Systematic pain assessment and treatment, Medication review, Organization of activities and Safety. We included baseline and 4-month follow-up data from a subgroup of nursing home patients who wore actigraphs (*n* = 107). The subgroup had different levels of blood pressure, from low (< 120) to high (≥ 141). Assessments included blood pressure, antihypertensive medication use, and sleep parameters as assessed by actigraphy.

**Results:**

We found a significant reduction in total sleep time at month four in the intervention group compared to the control group. When analysing the control group alone, we found a significant association between antihypertensive medication use and increased daytime sleep. We also found negative associations between blood pressure, antihypertensive medication use and sleep onset latency in the control group.

**Conclusions:**

Our results suggest a correlation between excessive daytime sleep and antihypertensive medication use. These findings should be followed up with further research, and with clinical caution, as antihypertensive medications are frequently used in nursing homes, and sleep problems may be especially detrimental for this population.

**Trial registration:**

The trial is registered at clinicaltrials.gov (NCT02238652).

## Background

Sleep problems, excessive sleepiness and daytime sleep are commonly observed in the nursing home population [1, 2]. A hallmark study by Jacobs et al., demonstrated that nursing home patients with dementia were neither awake nor asleep for a full hour throughout a 24-h period [3]. Although increased daytime sleep may be considered an adaptive response to impaired night-time sleep, it is well-known that naps negatively impacts sleep at night [1]. This pattern of daytime sleep and night-time wake-periods is common in nursing home patients and in people with dementia [4].

Today’s nursing home patients are frail and multimorbid; 80 % have dementia, and almost half the patients have cardiovascular diseases such as hypertension, heart failure, and atrial fibrillation [5]. There is inconclusive research regarding the potential benefits or unwanted side effects and risks of antihypertensive medication use in nursing home patients and people with dementia [6]. While some argue that treatment is beneficial regardless of age [7, 8], others are more concerned, highlighting risks of side effects and adverse effects [9, 10]. In patients with dementia, antihypertensive treatment is associated with orthostatic hypotension, falls, and increased anticholinergic burden [11].

Although sleepiness and nightmares are listed as common side effects in antihypertensive medications, no studies have investigated the association between such medications and night- and daytime sleep in a nursing home population or in people with dementia. Meanwhile, effects in the central nervous system have been associated with centrally acting antihypertensive medications. Early studies demonstrated that beta-blockers affect sleep architecture and maintenance, with increased awakenings associated with lipophilic beta-blockers, including propranolol [12]. In an early study by Gislason et al., including 4064 Swedish men, a significant risk of excessive daytime sleepiness was associated with hypertension, but not with the use of beta-blockers [13]. A study by Nicholson et al. found an increased risk of sleepiness in six adult males after antihypertensive treatment [14]. None of these studies included older individuals, or people with dementia. In addition, a recent review of the antihypertensive treatment in people with dementia did not identify any study investigating sleep disturbances other than sleep apnea in relation to the use of antihypertensive medication [15].

There has been demonstrated a link between cardiovascular disease and disturbed sleep in elderly populations [13, 16–18]. One Japanese prospective cohort study investigated the association between sleep and nocturnal blood pressure in 107 institutionalized people with dementia, while controlling for antihypertensive treatment [19]. The authors found an association between night-time sleep problems and impaired reduction in blood pressure, though they did not identify any moderating effects of antihypertensive medication use. In a study by Kostis and colleagues, they found a negative effect of antihypertensive medication use (propranolol) on total sleep time and sleep maintenance (waking after sleep onset) in adults with mild hypertension, but found no effects in an older population (60–78 years) [20].

Ageing is related to several changes in sleep, including reduced total sleep time and increased sleep fragmentation e.g., waking during the main sleep period. Consequently, it is also more common to observe daytime napping [21, 22]. These changes in sleep are complex and multicausal. They may partly be due to neurological changes, such as loss of cells in the “master clock”, superchiasmatic nuclei. Also, several conditions that are increasingly common in old age include symptoms that can disrupt sleep. This includes congestive heart failure and coronary artery disease [23, 24]. Sleep problems may share physiological mechanisms with high blood pressure. Problems with falling asleep, sleep fragmentation and waking up early have been related to a state of hyperarousal [25]. Having these sleep problems in combination with hyperarousal has been associated with a significant risk of hypertension [26]. In addition, the use of beta-blockers may inhibit pineal gland activity, resulting in suppressed levels of the melatonin during the night, which may negatively impact sleep [27].

In a recent study where we implemented a systematic medication review in nursing homes, we found that both nursing home patients with low and normal blood pressure (i.e., a systolic and diastolic blood pressure below 160 mmHg and 90 mmHg) used a median of one antihypertensive medication. Those with high blood pressure (a systolic and diastolic blood pressure above 160 mmHg and 90 mmHg) had a median of two antihypertensive medications. The medication review reduced the use of antihypertensive medications, leading to a temporarily increase in the systolic blood pressure. Meanwhile, by follow-up at month nine, the blood pressure had reached initial levels [28, 29].

Cardiovascular disease and antihypertensive medications may cause both day- and night-time symptoms affecting sleep. Consequently, it is necessary to investigate sleep parameters throughout a 24-h period. No study has investigated this association using 24-h, objective sleep assessment by actigraphy. This current study aimed to investigate the effect of a multicomponent intervention, including a systematic medication review, on day- and night-time sleep in a large nursing home population. In particular, we investigated the association between antihypertensive medication use, blood pressure and day- and night-time sleep at baseline and at 4 months follow-up.

## Methods

The current study was based on secondary analyses from the cluster randomized controlled COSMOS trial (cRCT). This was a multicomponent intervention trial, where the COSMOS acronym represents the intervention components: COmmunication, Systematic pain assessment and treatment, Medication review, Organization of activities, and Safety. The trial was conducted between May 2014 and December 2015. The published protocol describes the trial design, intervention, and sample size analyses in detail [30]. Related articles describe different aspects of the trial for instance the effect of the intervention on quality of life and neuropsychiatric symptoms [29], the effect of communication [31, 32], and the effect of the medication review [28, 33].

### Study design and participants

The COMSOS trial was an cRCT, lasting for four months, with data assessments at baseline, month four and a nine-month follow-up. We invited 765 patients from 72 units in 37 nursing homes from eight municipalities in Western and Eastern Norway to participate. For the actigraphy subproject, which provides data for the present study, 107 patients from both the intervention and control group were randomly selected from 19 nursing home units in four municipalities. Only nursing home unites with long-term care patients were included. Exclusion criteria were life expectancy < 6 months, patients < 65 years, and schizophrenia. In the present study, we used baseline and 4-month data, as there was no actigraphic measurements in either control or intervention group at the nine-month follow-up. In addition, patients suffering from any form of chronic upper body movement disorder or paralysis, and patients with less than 5 days and nights of actigraphy recordings, were excluded from the actigraphy subproject.

### Intervention

The multicomponent COSMOS intervention was implemented by a two-day educational seminar for all intervention units. The educational seminar included training and lectures in the multicomponent COSMOS intervention: Communication in the form of Advanced Care Planning, systematic pain assessment and treatment, medication review, organization of activities and safety. These units sent at least two nurses to the seminar who became that unit’s COSMOS ambassadors. In addition, the nursing home managers, physicians, and pharmacist were invited. The education program covered research-based knowledge about communication and advance care planning, pain assessment and treatment, multidisciplinary medication review, and organization of activities. After the seminar, the COSMOS ambassadors trained the rest of the staff in their units [34]. For the medication review, the COSMOS ambassadors and physicians each received their own written material, preparing them for the multidisciplinary medication review session together with two researchers (BSH and CG). Prior to the medication review, nurses, alongside the researchers assessed the patients with relevant clinical tools, and extracted the patients’ medication list, blood results, and diagnoses from the medical records. The physicians received a short description of the assessment tools used in the medication review, the Norwegian guidelines for medication reviews [35, 36], the STOPP/START version 2 criteria potentially inappropriate prescribing in older people [37], and an anticholinergic medications list [38].

### Outcome measures

Sleep was assessed with actigraphy, using the Philips Actiwatch Spectrum, which was worn on the patients’ dominant or mobile wrist continuously for 24 h throughout 7 consecutive days at baseline and month 4 [39, 40]. The actigraphs were placed on the dominant/mobile wrist to increase movement detection in this immobile population. The data was analysed with the Respironics Actiware 5 software, yielding the following standard sleep parameters: minutes of daytime sleep, minutes of sleep onset latency (time from going to bed until falling asleep), minutes of wake after sleep onset, minutes of early morning awakening (defined as minutes from waking up, until helped out of bed) and minutes of total sleep time. All variables were calculated as mean minutes per day/night for all patients with at least five valid days of actigraphy recording. Nursing home staff received both verbal and written instruction to push the event button on the actigraph at bed and rise times (light off in the night/light on in the morning).

In the sleep scoring protocol, rest intervals were set using a standardized hierarchical approach based on: (1) event markers, (2) light and activity data, and (3) light or activity data. Inter-scorer reliability was ensured by comparing 30 actigraphy recordings, scored by two independent scorers, in terms of total time in bed and total sleep time. Participants had to complete at least five-night recordings to be included. Sleep/wake status was scored for each one-minute epoch using the Actiware 6 software, with the sensitivity set to medium.

Medications registered in the patients’ medical records were coded according to Anatomical Therapeutic Chemical Index (ATC) classes [41]. The number of antihypertensive medications summed for each patient derived from the following five medication classes: C03C High Ceiling Diuretics (loop-diuretics), C07A Beta-blockers, C09A Plain Angiotensin-Converting-Enzyme Inhibitors, C09C Plain Angiotensin II Antagonists, and C08C Calcium Channel Blockers with mainly vascular effect. Blood pressure (measured in mmHg) and pulse was assessed in adherence with local procedure. Diagnoses were obtained from the participants’’ medical records.

Functioning in terms of cognitive function and activities of daily living, was assessed using the Mini Mental State Examiner (MMSE) and the Lawton and Brody Self-maintenance Scale. The MMSE assesses the level of cognitive impairment. Scores range from 0 to 30, with the following recommended cut-offs: 0–11 = severe dementia, 12–17 = moderate dementia, 18–23 = mild dementia, and 24–30 = no [42]. the Lawton and Brody Self-maintenance Scale includes six items (composite score range 0–30), where a lower value indicates better functioning and independence [43].

### Sample size analysis

The COSMOS trial’s power analysis was based on the primary outcome, quality of life. Based on the change expected, the sample size was estimated to 520 participants in total, factoring in cluster design and drop out. No posteriori analysis was performed for secondary analyses.

### Randomization and blinding

The included nursing home units were randomized to intervention groups or control groups (standard care) per participating municipality. Each unit was defined as a cluster and was randomized with a random number sequence in SPSS 18. The randomization was completed as a constrained complete list randomization stratified on 33 participating sites to ensure almost equal matched distribution to geographic and monetary status. This was a single-blinded study where the nursing homes were naïve to their allocation. When using interventions like staff education, with a clear focus on implementation, double-blinding is not feasible. Indeed, the single-blind design is described as most appropriate.

### Statistical analyses

Both the intervention and control group were included in descriptive analyses and baseline analyses, as well as in the analyses of the intervention effect. Because the intervention group received a multicomponent intervention, it would not be possible to exclude changes related to other components of the trial in the follow-up analyses. Using data from the control group allowed us to use that group as a population in an observational follow-up design. Baseline characteristics are described by mean and standard deviation (SD) for continuous normally distributed variables, median and inter quartile range (IQR) for continuously non-normally distributed variables and number of patients and percentages for categorical variables.

To determine the effect of the COSMOS intervention on the five different sleep parameters (daytime sleep, total sleep time, sleep efficiency, wake after sleep onset, and early morning awakening), we performed separate linear mixed models analyses for each outcome measure, with fixed effects for group, time and their interaction (i.e., the intervention effect), and random intercepts for patients, and nursing home unit if necessary.

To investigate baseline associations between sleep parameters and antihypertensive medication use or blood pressure, fifteen separate linear regression models were fitted. We performed both unadjusted analyses, and analyses adjusted for age, gender and hypertensive diagnoses at baseline (yes/ no). Blood pressure measurements, both systolic and diastolic, were divided by 10 to increase the readability of the coefficients, a change of 1 in the coefficient reflects a 10-mmHg change in blood pressure.

We used data from the control group to study associations between changes in sleep parameters and change in antihypertensive medication use or change in systolic or diastolic blood pressure from baseline to month 4. The analyses were linear mixed effect models with fixed effects for exposure (antihypertensive medication use, systolic or diastolic pressure) and time, and random intercepts for patients, and nursing home unit if necessary. Results are reported both unadjusted and adjusted for age, gender and hypertensive diagnoses at baseline.

For the regression models, clustering was accounted for with regards to daytime sleep. For all other outcomes, clustering had no added effect to the model, and the simplest model was retained.

Significance level was set to 0.05. Analyses were performed using STATA 16 (StataCorp, Texas, USA).

### Ethics, consent and permissions

All patients and their families were informed about the study, both orally and in writing. The information described the intervention, what data would be collected, and that data collected would be published in scientific publications. When patients lacked the capacity to consent, informed presumed consent was obtained in writing from the family. If the patients in the actigraphy subproject appeared to be bothered by the actigraph, staff was instructed to help them remove the watch. The COSMOS trial adheres to the Helsinki declaration and Norwegian law and has been approved by the Regional Ethics Committee West (2013/1765). The trial is registered at clinicaltrials.gov (NCT02238652).

## Results

Out of the 765 invited participants, the COSMOS trial included 545 patients from 67 nursing home units. The actigraphy subproject included 107 patients. Due to actigraph malfunction and missing data (less than 5 recorded days and nights), 17 were excluded leaving 90 participants with actigraphy protocols who were eligible to be included in our analyses.

At baseline, 52 % (*n* = 47) of the patients in the actigraph sub-group used antihypertensive medications. According to the diagnoses in the medical records, 2 % (*n* = 2) of the patients had elevated blood pressure, and 27 % (*n* = 24) had uncomplicated hypertension (Table 1). At month four, 11 patients in the intervention group and 5 in the control group had quitted using antihypertensive medications, while only one patient had started using these medications (from the intervention group).

**Table 1:** Baseline characteristics for nursing home residents in total, and by group

	Control group			Intervention group			Both groups		
	Mean/	(SD)/	*N*	Mean/	(SD)/	*N*	Mean/	(SD)/	*N*
	n/	(%)/		n/	(%)/		n/	(%)/	
	median	(IQR^a^)		median	(IQR^a^)		median	(IQR^a^)	
**Demographic variables:**									
Females	26	(67%)	39	43	(84%)	51	69	(77%)	90
Age	85.3	(7.6)	39	88.2	(8.3)	51	87.0	(8.1)	90
BMI	23.5	(4.6)	32	24.0	(3.9)	48	23.8	(4.2)	80
Systolic blood pressure^b^	128	(17)	37	130	(18)	49	129	(18)	86
Diastolic blood pressure^b^	70	(13)	37	70	(12)	49	70	(12)	86
Activities of daily living^c^	17.0	(5.5)	39	17.8	(.15)	51	17.3	(5.3)	90
Regular prescriptions	7.5	(4.0)	39	7..6	(3.9)	51	7.6	(3.9)	90
Diagnoses	4.2	(2.0)	39	4.5	(2.9)	51	4.4	(2.6)	90
**Cognitive function:**									
MMSE^a^ (0-30)	12	(7-17)	35	10	(5-13)	49	11	(5-16.5)	84
No (24-30)	2	(6%)		3	(6%)		5	(6%)	
Mild (18-23)	6	(17%)		7	(14%)		13	(15%)	
Moderate (12-17)	10	(28%)		10	(21%)		20	(24%)	
Severe (0-11)	17	(49%)		29	(59%)		46	(55%)	
**Blood pressure**^b^**:**									86
Low (blood pressure < 120)	12	(32%)		12	(24%)		24	(28%)	
Normal (120 ≤ blood pressure < 141)	18	(49%)		25	(52%)		43	(50%)	
High (blood pressure ≥ 141)	7	(19%)		12	(24%)		19	(22%)	
**Hypertension diagnoses:**									
Elevated blood pressure (K85)	1	(3%)	39	1	(2%)	51	2	(2%)	90
Uncomplicated hypertension (K86)	11	(28%)	39	13	(25%)	51	24	(27%)	90
Hypertension with organ complications (K87)	1	(3%)	39	2	(4%)	51	3	(3%)	90
**Medication (ATC Code):**									
Antihypertensive medications	21	(54%)	39	26	(51%)	51	47	(52%)	90
High-ceiling diuretics (C03C)	13	(33%)	39	13	(25%)	51	26	(29%)	90
Beta-blockers (C07A)	13	(33%)	39	14	(27%)	51	27	(30%)	90
Plain angiotensin II antagonists (C09C)	3	(8%)	39	9	(18%)	51	12	(13%)	90
Plain ACE inhibitors (C09A)	3	(8%)	39	2	(4%)	51	5	(6%)	90
Calcium channel blockers, mainly vascular effect (C08C)	2	(5%)	39	5	(10%)	51	7	(8%)	90
**Sleep**^a^**:**									
Total sleep time (TST)	443	(348-552)	37	514	(425-601)	46	492	(371-572)	83
Sleep onset latency (SOL)	24	(10-95)	37	23	(10-64)	46	24	(10-79)	83
Wake after sleep onset	149	(105-193)	37	127	(82-202)	46	142	(93-202)	83
Daytime sleep (DTS)	170	(102-237)	37	115	(204-342)	45	180	(110-276)	82
Early morning awakening (EMA)	39	(31-73)	37	28	(14-56)	46	35	(19-70)	83

Data are presented as mean (SD), number (%) or median (IQR), with score ranges in parentheses

^a^Inter Quartile Range

^b^Measured in mmHg

^c^Measured on a scale from 0 to 30, where 0 is independent and 30 is totally independent in activities of daily living

^d^Higher scores indicate better cognitive function

^e^Reported as average minutes per day/night

Looking at both groups combined at baseline, we found no significant associations between sleep parameters and blood pressure or antihypertensive medication use (Table 2, systolic data shown).

**Table 2:** Associations between sleep parameters and blood pressure or antihypertensive medication use at baseline, for both groups combined

Outcome^a^	Exposure	Unadjusted association (95 % CI)	***p***-value	Adjusted association^b^ (95 % CI)	***p***-value	N
Total sleep time	Anti hypertensives^c^	-1.4 (-71.7 – 69.0)	0.97	14.1 (-63.7 – 91.9)	0.72	83
Total sleep time	Systolic blood pressure^d^	-3.6 (-24.2 – 17.1)	0.73	-3.1 (-24.9 – 18.7)	0.78	79
Sleep onset latency	Anti hypertensives^c^	-11.6 (-46.7 – 23.6)	0.53	-20.9 (-59.6 – 17.8)	0.29	83
Sleep onset latency	Systolic blood pressure^d^	-0.9 (-11.2 – 9.3)	0.86	-2.4 (-13.1 – 8.3)	0.66	79
Wake after sleep onset	Anti hypertensives^c^	-14.9 (-50.0 – 20.3)	0.40	-12.0 (-49.8 – 25.7)	0.53	83
Wake after sleep onset	Systolic blood pressure^d^	4.5 (-5.8 – 14.9)	0.39	4.3 (-6.2 – 14.7)	0.42	79
Daytime sleep	Anti hypertensives^c^	22.7 (-29.6 – 75.1)	0.39	31.8 (25.9 – 89.5)	0.28	82
Daytime sleep	Systolic blood pressure^d^	-7.0 (-22.0 – 8.2)	0.36	-6.7 (-22.7 – 7.4)	0.41	78
Early morning awakening	Anti hypertensives^c^	-10.6 (-39.8 – 18.6)	0.47	-7.0 (-38.8 – 24.8)	0.67	83
Early morning awakening	Systolic blood pressure^d^	-4.9 (-13.1 – 3.4)	0.24	-3.9 (-12.5 – 4.7)	0.37	79

^a^Reported as average minutes per day/night

^b^Adjusted for age, gender and hypertensive diagnoses at baseline (yes/ no)

^c^Anti-hypertensive users compared to non-anti-hypertensive users

^d^Increase in sleep parameter associated with 10 mmHg increase in systolic blood pressure

Interestingly, looking at the intervention effects of the COSMOS study on sleep parameters, there was a significant reduction in total sleep time at month four in the intervention group as compared to the control group (mean difference in change (MC) = -58.5 min, 95 % Confidence Interval (CI)=-115.5 – -1.6, *p* < 0.05), with a corresponding reduction within the intervention group (42.5 min, CI=-76.6 – -8.4, *p* < 0.05). There were no other significant changes in sleep parameters from baseline to month four (Table 3).

**Table 3:** Estimated intervention effects, and changes within groups from baseline to month four, for five different sleep parameters

Outcome^a^	Within-group change		Intervention effect	***p***-value^**b**^	***n***
	Control	Intervention			
	(95 % CI)	(95% CI)	(95 % CI)		
Total sleep time	16.0 (-29.6 – 61.6)	-42.5* (-76.6 – -8.4)	-58.5* (-115.5 – -1.6)	0.04	90
Sleep onset latency	-7.2 (-33.4 – 18.7)	8.3 (-11.2 – 27.9)	15.6 (-16.9 – 48.2)	0.35	90
Wake after sleep onset	2.9 (-22.9 – 28.8)	18.3 (-1.1 – 37.7)	15.4 (-17.0 – 47.7)	0.35	90
Daytime sleep	10.6 (-20.9 – 42.2)	3.1 (-20.7 – 27.0)	-7.5 (-47.1 – 32.0)	0.71	90
Early morning awakening	-24.3 (-49.5 – 1.0)	18.3 (-1.1 – 37.7)	28.6 (-3.1 – 60.4)	0.07	90

^a^Reported as average minutes per day/night

^b^*p*-value for intervention effect

*p < 0.0

Looking at the observational follow-up data in the control group, we found a significant association between increased systolic blood pressure and increased total sleep time from baseline to month 4 (MC 25.0 min, CI = 4.5–45.5, *p* < 0.05) (Table 4). There were significant negative associations between sleep onset latency and antihypertensive medication use (MC -51.1 min, CI=-95.6 – -7.4, *p* < 0.05) and systolic blood pressure (MC -13.7 min, CI=-22.0 – -5.4, *p* < 0.05). Thus, antihypertensive medication use was associated with a shorter sleep onset latency. Similarly, increased systolic blood pressure was associated shorter sleep onset latency. Importantly, there was an association between antihypertensive medication use and increased daytime sleep within the control group from baseline to month 4 (MC 50.3 min, CI = 4.8–95.6, *p* < 0.01). There were no significant associations between diastolic blood pressure and the sleep parameters. Changes in antihypertensive medication use and systolic pressure are presented in Tables 5 and 6.

**Table 4:** Associations between change in sleep parameters and change in blood pressure or change in antihypertensive medication use from baseline to month 4, in the control group

Outcome^a^	Exposure	Unadjusted association (95 % CI)	***p***-value	Adjusted association^b^ (95 % CI)	***p***-value	n
Total sleep time	Anti hypertensives^c^	15.6 (-74.0 – 105.2)	0.73	24.0 (-71.5 – 119.5)	0.62	39
**Total sleep time**	**Systolic blood pressure**^**d**^	**23.5 (3.2 – 43.8)**	**0.02**	**25.0 (4.5 – 45.5)**	**0.02**	**38**
**Sleep onset latency**	**Anti hypertensives**^**c**^	**-44.8 (-86.5 – -3.1)**	**0.04**	**-51.1 (-95.6 – -7.4)**	**0.02**	**39**
**Sleep onset latency**	**Systolic blood pressure**^**d**^	**-12.7 (-20.9 – -4.4)**	**0.01**	**-13.7 (-22.0 – -5.4)**	**0.00**	**38**
Wake after sleep onset	Anti hypertensives^c^	-8.7 (-49.5 – 32.0)	0.67	-12.7 (-56.0 – 30.6)	0.57	39
Wake after sleep onset	Systolic blood pressure^d^	-3.7 (-13.9 – 6.5)	0.48	-2.8 (-13.1 – 7.6)	0.60	38
**Daytime sleep**	**Anti hypertensives**^**c**^	**61.0 (4.3 – 117.8)**	**0.035**	**50.3 (4.8-95.6))**	**0.03**	**39**
Daytime sleep	Systolic blood pressure^d^	3.7 (-8.4 – 15.8)	0.55	1.9 (-10.1 – 13.9)	0.76	38
Early morning awakening	Anti hypertensive^c^	-20.4 (-63.2 – 22.3)	0.35	-13.1 (-59.0 – 32.8)	0.58	39
Early morning awakening	Systolic blood pressure^d^	-9.6 (-20.2 – 1.0)	0.08	-9.1 (-19.9 – 1.7)	0.1	38

^a^Reported as average minutes per day/night

^b^Adjusted for age, gender and hypertensive diagnoses at baseline (yes/ no)

^c^Anti-hypertensive users compared to non-anti-hypertensive users or change in anti-hypertensive medication use from baseline to month 4 for a patient

^d^Increase in sleep parameter associated with 10 mmHg increase in systolic blood pressure, the association can be interpreted as between-patients association or within-patient association

**Table 5:** Changes in antihypertensive medication use from baseline to month 4, by group

Antihypertensive medication use	Control	Intervention
Never used^a^	18	24
Starters^b^	0	1
Quitters^c^	5	11
Stable users^d^	16	15

^a^Did not use at baseline or at month 4

^b^Did not use at baseline, user at month 4

^c^User at baseline, but not at month 4

^d^User at both baseline and month 4

**Table 6:** Changes in blood pressure from baseline to month 4, control group

	Control Mean (SD)	
	Baseline *N*=37	Month 4 *N*=27
Systolic pressure, mmHg	128 (17)	123 (16)

## Discussion

Our primary aim was to investigate the association between antihypertensive medication use, blood pressure and day- and night-time sleep. Alongside our multicomponent intervention including a systematic medication review, we found a significant reduction in total sleep time at month 4 in the intervention group compared to the control group. Further, a significant association between increased systolic blood pressure and increased total sleep time were demonstrated, from baseline to month 4 in the control group. Both increasing blood pressure and antihypertensive medication use were associated with reduced sleep onset latency. Antihypertensive medication use was associated with increased daytime sleepiness. These findings are of key importance for the clinician because daytime sleep and nighttime sleep problems are common in nursing home patients of whom 50 % are treated with antihypertensives despite almost 80 % having low or normal blood pressure.

Over half of the patients in the actigraphy subgroup received antihypertensive medications, at baseline, which illustrates how common the use of such medications is in this population and thus also the clinical urgency of investigating potential side effects. Sleep problems have been related to severe health consequences; clinicians who prescribes and reviews the medications in this population should therefore consider the effects in this study carefully. The results also call for further investigation with a study design that includes sleep and antihypertensive medication use as the primary point of investigation.

We have previously demonstrated a significant higher deprescribing of antihypertensive medications in the intervention compared to the control group [28]. In that publication, also based on COSMOS data, the intervention group showed an increase in blood pressure when antihypertensive medications were reduced/withdrawn, from baseline to month four (see publication for full details on changes in medication and effects on blood pressure). It is possible that these changes also affected their sleep, although research is lacking to confirm this hypothesis. In addition, the medication review may have resulted in the discontinuation of several medications that impact sleep, such as antidepressants and hypnotics. Indeed, the reduced total sleep time in the intervention group compared to control may in part be a result of a withdrawal-effect. In our previous study on medication review, the blood pressure returned to baseline values from month four to month nine. Unfortunately, we have no actigraphy recordings for the nine-month follow-up, as it would be interesting to investigate whether the sleep improved in the intervention group in conjunction with the normalization of blood pressure.

It is also possible that changes in the clinical practice in the intervention units had an impact on the reduced total sleep time. In our education of the intervention units, we communicated that it may be disadvantageous to stay in bed longer than intended sleep time. Because actigraphy is sensitive to detect sleep but show less specificity for wakefulness, a substantially reduced time in bed could impact total sleep time. In order to investigate the longitudinal associations between antihypertensive medication use and sleep without the contamination of our multicomponent intervention we also analysed the effects in the control group alone.

An important finding was the association between antihypertensive medication use and increased daytime sleep. This may indicate that the medication has a negative central nervous impact on these patients, affecting their wakefulness. Indeed, previous studies show that beta-blockers is associated with an increased incidence of daytime fatigue [20]. Fatigue and sleepiness are not identical symptoms, and the results may thus be interpreted slightly differently. It is possible that the antihypertensive medications led to increased lethargy and less movement. This would have been interpreted as sleep by an actigraphy. Alternatively, the medication use may have caused an increased propensity to sleep during the day. The latter, in particular would have affected the subsequent night-time sleep. Our group also investigated the association between low systolic blood pressure and neuropsychiatric symptoms in people with dementia using antihypertensive medication [33]. However, in this study, no difference between high and low systolic blood pressure and the symptom clusters were identified.

There was a negative association between sleep onset latency and antihypertensive medication use, which could be interpreted in relation to the daytime sleep findings. Daytime sleep is related to reduced build-up in sleep need, which is subsequently related to difficulties falling asleep and reduced deep sleep [44]. A downward spiral may thus be established, where extended daytime naps lead to difficulties falling asleep and poor night-time sleep quality, which in turn increases the urge to nap during the day. Conversely, the significant association between sleep onset latency and high blood pressure, may be due to other mechanisms. It could have been expected that high blood pressure was related to a general higher level of arousal [25, 26]. Since blood pressure were not related to daytime sleepiness, this could be the case, but the decreased sleep onset latency does not confirm this hypothesis. A study designed to review the effects of BP and antihypertensive medication use on central sleep parameters are needed to fully understand these effects.

There may be seasonal variations in sleeping patterns in nursing home patients. The COSMOS trial started the data collection during the summer, and the data collection continued throughout the following year, starting up in different municipalities in a stepwise manner. If all participants had started in the summer, the effect of the intervention on sleep could have been affected by the reduced light during winter. Meanwhile, with different startup times, seasonality is less likely to represent a bias in our dataset.

### Strengths and limitations

There are some limitations to this study. We relied on the local methods for measuring blood pressure in each unit, which resulted in no standard procedure for assessment. Antihypertensive medications were often prescribed prior to nursing home placement; thus, we did not have reliable data on when and why antihypertensive medications were prescribed. The number of patients who both used antihypertensive medications, and who were included in the actigraphy subprojects was low, and we cannot exclude the possibility of type 2 error. Our study population was representative for the nursing home population in general, i.e., polypharmacy was common with a mean use of over 7.6 regular medications. These medications often include medications such as antidepressants, opioids, and hypnotics which also affects sleep [45]. Thus, we cannot rule out the effects of other medications, or the discontinuation of these medications. Meanwhile, by investigation the longitudinal effects in the control group only, we excluded any effects related to the COSMOS medication review, and multicomponent intervention. Meanwhile, this observational design introduces an increased risk of confounding variables affecting the results. We did not correct for multiple testing in this study. We performed relatively few single variable regression analyses, including few variables in the same analysis, which could arguably make the need for correction less pressing. Together with the fact that we had a relatively low n in this study, the risk of a type 2 error would be high.

## Conclusions

Antihypertensive medication use was frequent in our population of nursing home patients. Our results suggest a correlation between excessive daytime sleep and hypertensive medication use, which also can impact the night-time sleep. These findings are important, and needs to be further investigated, since antihypertensive medications are frequently used in nursing home patients, and sleep problems may be especially detrimental for this population.

## Data Availability

The dataset analysed during the current study are not publicly available due restrictions made by the ethical committee for safe data storage, but may be available upon reasonable request made to the corresponding author.

## Abbreviations

ATC: Anatomical Therapeutic Chemical Index
BP: Blood Pressure
CI: Confidence Interval
COSMOS: COmmunnication, Systematic pain assessment and treatment, Medication Review, Organization of activities, Security
IQR: Inter Quartile Range
MC: mean difference in change
RCT: Randomized Controlled Trial
SD: Standard Deviation
START 2: Screening Tool to Alert to Right Treatment
STOPP 2: Screening Tool of Older Persons’ Prescriptions
